# Management of dental anxiety via distraction technique

**DOI:** 10.4317/jced.57660

**Published:** 2021-04-01

**Authors:** Job Torres-Gomez, Stephen C. Arnason, Wyeth L. Hoopes, Kraig S. Vandewalle

**Affiliations:** 1 DMD, MS. Capt, USAF, DC. Deputy Program Director. Advanced Education in General Dentistry Residency. Air Force Postgraduate Dental School. Uniformed Services University of the Health Sciences. 2501 Capehart Rd. Offutt, Air Force Base, Bellevue, NE, USA; 2 DDS, MS. Maj, USAF, DC. Training Officer. Advanced Education in General Dentistry Residency. Air Force Postgraduate Dental School. Uniformed Services University of the Health Sciences. 1615 Truemper St. Joint Base San Antonio - Lackland, TX, USA; 3 DDS, MS. Col (ret), USAF, DC. Director of Dental Research. Advanced Education in General Dentistry Residency. Air Force Postgraduate Dental School. Uniformed Services University of the Health Sciences. 1615 Truemper St. Joint Base San Antonio - Lackland, TX, USA

## Abstract

**Background:**

The purpose of this study was to evaluate the use of a stress ball as a distraction technique on stress levels of patients undergoing a dental procedure.

**Material and Methods:**

A randomized, split-mouth design was conducted using 20 adult subjects requiring scaling and root planing (Sc/RP) in all four quadrants. Each side of the mouth (maxillary/mandibular) received Sc/RP with local anesthetic with or without the use of a stress-ball distraction over two separate sessions. Subjects completed two pre-procedural questionnaires (Spielberger State-Trait Anxiety Inventory, STAI; Modified Dental Anxiety Scale, MDAS) before and after each treatment session. A Galvanic Skin Response (GSR) sensor (Neulog) was used throughout each session to measure skin conductance or sweat.

**Results:**

No significant difference in GSR scores was found during treatment with or without the use of the stress ball. Also, no significant differences in the change in STAI or MDAS scores were found with or without the use of a stress ball.

**Conclusions:**

The results of this study found that the use of a stress ball as a distraction technique did not result in any significant reduction in stress levels in subjects undergoing scaling and root planing with local anesthesia.

** Key words:**Anxiety, distraction, stress ball.

## Introduction

Dental anxiety is a constant challenge for both the practitioner and patient that often impairs the ability of the dentist to deliver routine care. Anxiety of dental treatment stems from a multitude of factors, including traumatic past dental experiences (1). Often stories portrayed through media or word of mouth convey a disturbing portrayal of dentistry. People also report the lack of understanding and a sense of vulnerably, specifically associated with lying in the supine position, as a cause for apprehension. Additionally, proprioceptive triggers such as smells, sounds, sight, and touch are a common source of fear (2). One study reported that the sight and sounds of the dental drill produces panic in some patients (3). Another example cites eugenol as a scent that commonly triggers dental post-traumatic stress (4). All these nociceptive feelings can result in the creation of patients with the inability to cope with preventative care. Studies have shown that anxiety has a direct impact in decreasing the pain threshold, and has been implicated in elevating pain intensity (5,6). This psychological fear can drive patients to avoid regular dental care as stated by Coriat “any dental surgery, no matter how minor, or even dental prophylaxis, may be so postponed or procrastinated that the inroads of disease may affect the entire dental apparatus (7).”

Cognitive refocusing is a method based on theory of pain where a distraction diverts pain perception by focusing attention to more amusing attractions. In this method, pain perception is reduced due to the increased mental demand towards more pleasant stimuli (8). A range of distraction methods that are known to work in decreasing patient anxiety and thus perceived pain perception include music, audiovisual, and touch (9). The use of music and video distraction techniques have been demonstrated to be statistically significant in reducing dental anxiety (10,11). Another method of distraction, employing stress balls, may be a more simple, yet cost effective approach to cognitive refocusing. In medical settings, the use of stress balls as a method of reducing patient anxiety and perceived pain has been evaluated with equivocal results (12,13). However, the application of stress balls as a touch-distraction method has yet to be evaluated in a dental setting.

The purpose of this study was to evaluate the use of stress balls as a distraction technique and determine how it affects stress levels of patients undergoing routine scaling and root planning procedures under local anesthetic. The null hypothesis tested was that there would be no difference in anxiety assessments and galvanic skin response during scaling and root planing with local anesthesia, with or without the use of stress balls.

## Material and Methods

**Figure 1:**
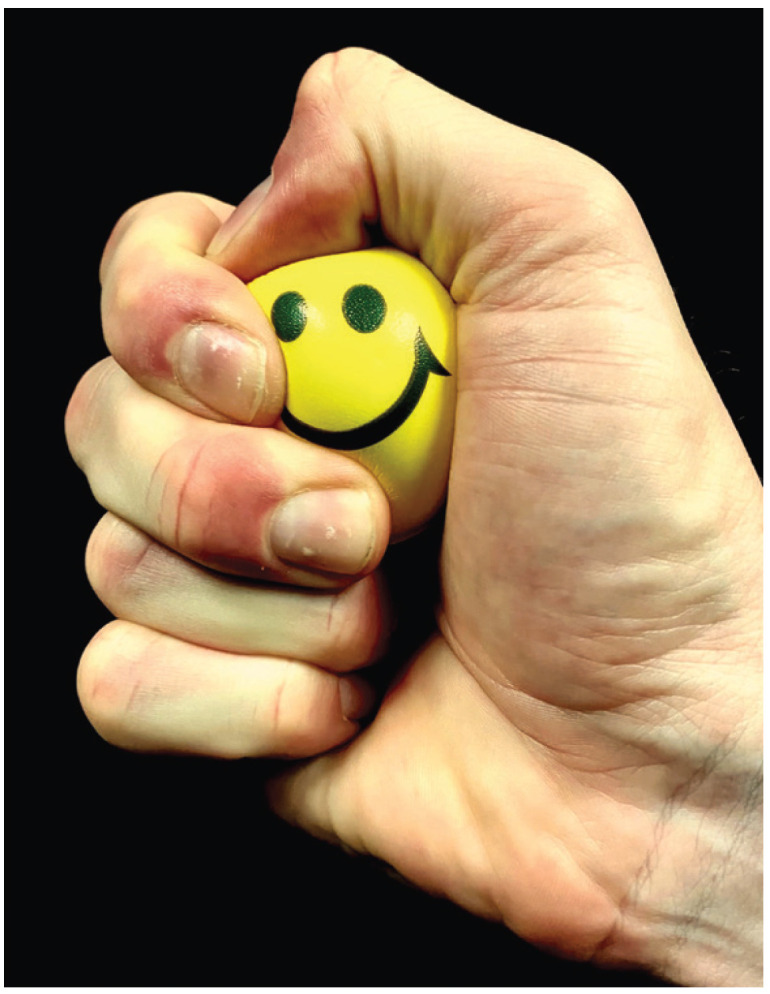
Patient squeezing stress ball with dominant hand.

**Figure 2:**
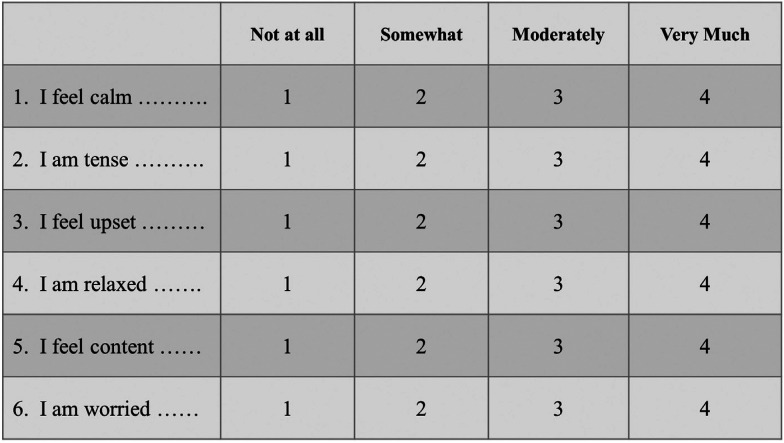
Six-item short-form Spielberger State-Trait Anxiety Inventory (STAI).

**Figure 3:**
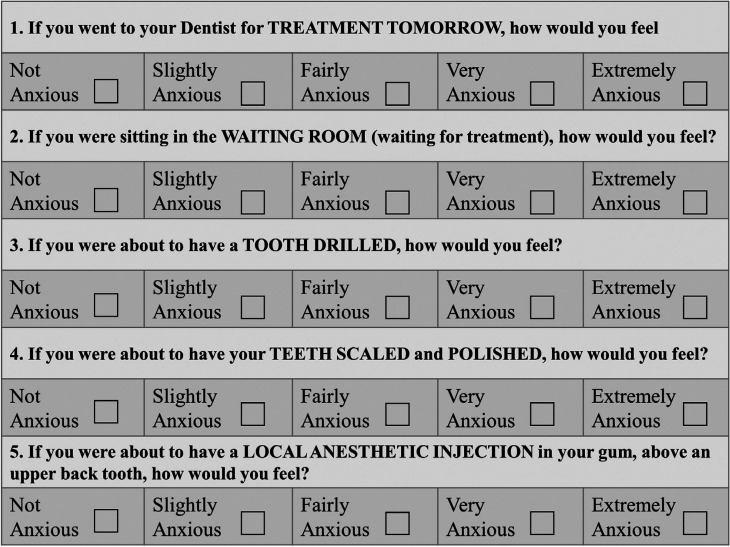
Modified Dental Anxiety Scale (MDAS).

The Institutional Review Board at Wilford Hall Ambulatory Surgical Center, Joint-Base San Antonio, Lackland, TX, USA approved this protocol (#FWH 20190044H). The subject population for this study was a random selection of active duty or Department of Defense beneficiaries over the age of 18 with mild, moderate, or severe periodontitis requiring scaling and root planing (Sc/RP) in all four quadrants with local anesthetic. This study used a randomized “split-mouth” design with one side of the mouth (maxillary and mandibular) receiving Sc/RP with local anesthetic with the use of a squeeze ball (Fig. 1) distraction (Why Worry? Be Happy! Neon Yellow Funny Face Stress Ball, Neliblu, Seattle, WA, USA) at one of two appointments (experimental group). The contralateral side (maxillary and mandibular) received Sc/RP with local anesthesia without the use of squeeze ball distraction (control group). Using a random number generator, treatment (with/without squeeze balls) and order (left vs. right side) were randomized per appointment visit. In order to evaluate patient’s anxiety, continuous Galvanic Skin Response (GSR) was used in combination with the six-item short form of the Spielberger State Trait Anxiety Inventory (STAI) and the Modified Dental Anxiety Scale (MDAS) assessments. Subjects completed a six-item short form Spielberger State-Trait Anxiety Inventory (STAI) (Fig. 2) and a Modified Dental Anxiety Scale (MDAS) (Fig. 3) before and after each procedure. STAI was originally established in 1970 as an assessment to allow patients to self-evaluate their current level of anxiety (14). The MDAS is a self-reported assessment consisting of 5 questions that evaluates dental anxiety. Created in 1995, the MDAS is a simplified version of the Corah’s Dental Anxiety Scale that makes it easier for patients to comprehend and faster to complete (15).

**Figure 4:**
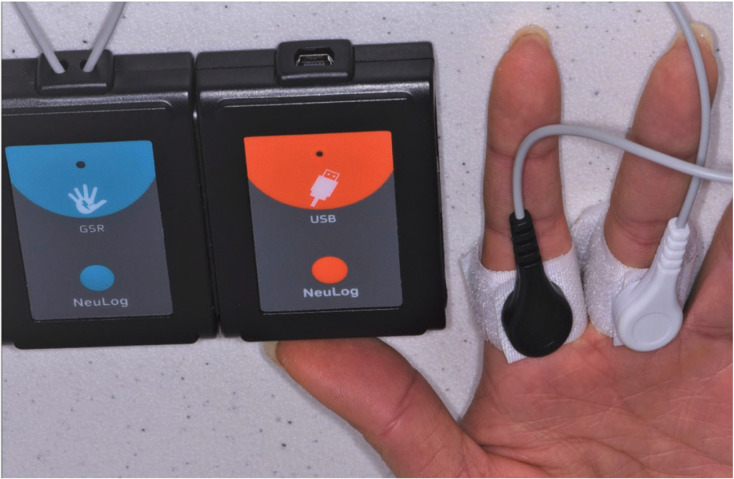
Velcro connectors were wrapped around two different fingers on the hand opposite the hand using the stress ball.

The Galvanic Skin Response monitor (Neulog Galvanic Skin Response, Carolina Biological Supply Company, Burlington, NC, USA) was used during each clinic session to measure skin conductance or sweat. Sweat creates a low-resistance path enabling the measurement of electric current. The conductivity of our skin changes according to unconscious emotional effects such as sudden noise, smell, touch, or pain. Higher GSR values are directly correlated to higher anxiety situations (16). Velcro connectors were wrapped around two different fingers on the hand opposite the hand using the stress ball (Fig. 4). Data were collected using Neulog 3 GSR software with a one-hour run time and a sampling frequency of 5 microsiemens (µS) per second. Monitoring with GSR and use of the distraction stress ball began when the patient was seated in the dental chair and ended with the completion of the procedure. The patient was instructed to squeeze the stress ball at any time during the procedure. Using a split-mouth design, the subjects served as their own control and a total of twenty subjects achieved a power of 80% to detect an 0.67 standard deviation difference or medium effect size for a two-sided test with a significance level of 0.05 (NCSS) PASS v.11.0.8, Kaysville, UT, USA.

A topical anesthetic (benzocaine 20% gel Topex, Sultan Healthcare, NJ, USA) was used to anesthetize the surface tissue at the sites receiving the injections for 2 minutes. Inferior alveolar and long buccal nerve block injections were administered for injections on the mandible and buccal infiltration injections were provided on the maxillary arch. The principle investigator performed the site injections to standardize the anesthetic flow rate and technique. The inferior alveolar nerve block injection was given at the pterygotemporal depression. The long buccal nerve block injection was given between the distal mandibular alveolar crest and the external oblique ridge. The maxillary infiltration injections were given in the buccal vestibule near the facial surfaces of the maxillary first premolar and first molar teeth. The anesthetic solution (2% lidocaine with 1:100,000 epinephrine, Henry Schein, Melville, NY, USA) was administered using a 27-gauge long needle (Monoject, Covidien AG, Neuhausen am Rheinfall, Switzerland) for the mandibular injections and a 30-gauge short needle (Monoject) for the maxillary injections, with a standard dental anesthetic syringe. A new needle was used for each injection site to ensure a fresh, sharp cutting tip and to control for injection site pain. Sc/RP was performed using hand and ultrasonic scalers Piezon Master 700, Hu-Friedy, Chicago, IL, USA).

Post treatment STAI and MDAS assessments were completed after every session. The STAI evaluation gives each item a weighted score from 1-4, with a range of scores from 20-80 (Fig. 2). Anxiety items (tense, upset, worried) carry a normal scoring trend with a value of 4 indicating a high level of anxiety. Anxiety absent items (calm, relaxed, content) are reversed, and values marked as 1, 2, 3, 4 are scored as 4, 3, 2, 1 respectively. All six scores are summed up and multiplied by 20 and divided by 6. According to Bekker *et al.*, a score range of 34-36 is considered normal (17). Evaluation of MDAS was completed using the MDAS Scale, where each item was scored from “not anxious” to “extremely anxious”, with an assigned numerical value from 1-5 respectively. The sum of all five items can range from 5-25, with a score of 11 – 14 considered moderate anxiety and over 15 considered high anxiety (15).

A median and interquartile range was determined per group for GSR and before and after treatment for both STAI and MDAS. A subgroup analysis of GSR, STAI, and MDAS scores was conducted on subjects initially reporting moderate to high anxiety before treatment with and without the use of the stress ball. Also, the change in STAI and MDAS scores before and after treatment were determined for each group. Data were evaluated using a Wilcoxon Signed Rank Test (alpha=0.5) with statistical software (SPSS, version 25, IBM, Armonk, NY). Additionally, subjects were asked after the second appointment to indicate whether they had preferred the first session, or the second session or if they had no preference between either sessions.

## Results

The subject pool was made up of 15 males and 5 females with ages ranging from 24-85 years (mean 54 years). The continuous GSR data were analyzed with a Shapiro Wilk test and found to be not normally distributed. Therefore the paired data were analyzed with a Wilcoxon Signed Rank Test. No significant difference in GSR scores (*p*=0.14) was found during treatment with or without the use of the stress ball. The use of the stress ball resulted in a median GSR of 0.80 and IQR of 0.77 µS, which was not significantly different than no use of the stress ball with a median GSR of 0.74 and IQR of 0.83 µS. Also, no differences in the change of STAI or MDAS scores were found with or without the use of a stress ball (*p*>0.05). See Table 1. Of the eight subjects reporting with pre-procedural STAI scores of high anxiety or nine subjects reporting with pre-procedural MDAS scores of moderate to high anxiety, no differences were found in a subgroup analysis with or without the use of the stress ball (*p*>0.05). See Table 2. Additionally, a subgroup analysis of GSR scores during treatment on six subjects that had higher levels of anxiety with both MDAS and STAI found that the use of the stress ball resulted in a median GSR of 0.80 and IQR of 1.06 µS, which was not significantly different (*p*=0.47) than no use of the stress ball with a median GSR of 0.75 and IQR of 0.68 µS. Of the 20 subjects participating in this study, 20% preferred the use of the stress ball, 35% preferred not to use the stress ball, and 45% had no preference.

**Table 1:**
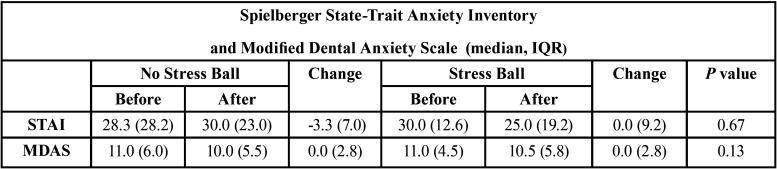
Results of the Spielberger State-Trait Anxiety Inventory (STAI) and Modified Dental Anxiety Scale (MDAS) of all subjects. Sample size equals twenty subjects for both STAI and MDAS.

**Table 2:**
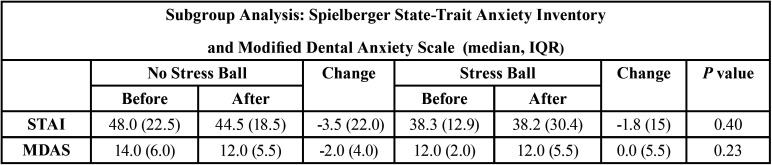
Results of the subgroup analysis of Spielberger State-Trait Anxiety Inventory (STAI) and Modified Dental Anxiety Scale (MDAS) of subjects reporting higher pre-procedural anxiety. Sample size equals eight subjects for STAI and nine subjects for MDAS.

## Discussion

Pain has been reported as a multidimensional experience, influenced by multiple interactions (18). Tracey and Mantyh described the perception of pain as a cognitive-evaluative, motivational-affective, and sensory-discriminative experience (19). Legrain *et al.* described a hypothesis where in a “neurocognitive model of attention, pain perception could be decreased by increasing the cognitive load.” In that model, pain sensation is decreased by an attention-grabbing task that increases the demand of attention away from the pain source (20). However, in this study, there was no difference in anxiety assessments and galvanic skin response during scaling and root planing with local anesthesia, with or without the use of stress balls in both the random population of subjects and the subgroup analysis of subjects reporting higher pre-procedural anxiety levels. Therefore, the null hypothesis was not rejected.

A study looking at the ability of children to reduce the stress of venipuncture through the use of stress balls showed no significant results. However, the average age was only nine years old and the procedure was a blood draw, two factors which may have limited the success of the technique (12). Another study involved the use of distraction techniques to include video, stress balls, music, conversation, or treatment as usual (control). The study examined adult subjects during minimally invasive venus surgery under local anesthetic. Similar to this study, a pre- and post-procedural STAI was utilized to evaluate levels of anxiety. The authors also utilized a pain questionnaire and a numeric rating scale to assess intraoperative pain and anxiety. The results of their study demonstrated that conversation, watching a video, or the use of stress balls significantly reduced patient stress and anxiety during the procedure (13). However, unlike this study, the researchers did not evaluate galvanic skin response to objectively measure patient stress and anxiety. In addition, this study utilized a within-subject design with subjects serving as their own control, potentially increasing the power to detect the effect of the use of stress balls on stress and anxiety.

The efficacy of GSR has been evaluated in multiple studies. In a 2016 literature review by Appukutan, it was concluded that “An extremely accurate objective method used in various studies to measure dental anxiety is galvanic skin response (10).” Caprara *et al.* conducted a study that demonstrated that GSR had a statistical significant correlation to dental anxiety. In that study, the data identified that the highest anxiety levels were a result of the local anesthetic injection (21). In another study evaluating GSR’s predictability in measuring children’s dental anxiety, 151 children from ages 5-7 were screened and confirmed for dental anxiety with a modified dental anxiety scale (MDAS), then subjected to simple dental restorative procedures while undergoing GSR monitoring. The results demonstrated that GSR was correlated to the pre-procedural MDAS evaluation and was a statistically significant method in evaluating children’s dental anxiety. Furthermore the use of heart rate for objective measurement was ruled out due to the potential side effects of the epinephrine, such as an epinephrine rush, causing an increase in heart rate as a direct effect of the epinephrine and not the patient’s anxiety (16).

Several studies have evaluated the legitimacy of the MDAS and STAI (22-27). One of the largest studies evaluating MDAS took place in 2000 with a sample size of 800 patients from 3 different countries - Northern Ireland, Finland, and Dubai. The patients attending dental clinics were handed a questionnaire booklet and then followed up with an invitation to participate in the study. The results demonstrated that the MDAS had high levels of consistency and validity (23). The original STAI contained 40 items and was extensively used in medical settings (24). In a study by Marteau and Bekker, the 40 items were shortened to only six items and produced correlation coefficients greater than 0.90 (25). In a follow up study, Tluczek *et al.* revisited the six-item short form SATI created by Marteau and Bekker and assessed 288 subjects at 2, 6, and 12 months. The short form was highly correlated with the 40-item STAI score, and all internal consistency reliabilities were greater than 0.90 (26). The effects of pre-procedural MDAS and STAI on patient anxiety levels has also been studied. In a study conducted by Humphris and Hull, the use of pre-procedural anxiety assessments did not increase patient anxiety (27).

This was the first study evaluating the use of stress balls as a distraction technique in a dental setting. Limitations to this study include the inability to control factors such as rate of injection administration, extent of probing depths, and severity of periodontal disease. Some subjects commented that the stress ball was either too firm or too large or small. These concerns can be a limiting factor since they could prevent the patient from actively engaging in the use of the stress ball. One subjective finding to note was that 30% of subjects did indicate that they thought the stress ball helped decrease anxiety during administration of local anesthetic. Additionally, of the ten subjects that used the stress ball in the first session, two requested the use of the stress ball at the subsequent appointment even though they were not randomized to use it. Future studies could evaluate the use of the stress ball only during the administration of local anesthetic or include the use of more stressful dental treatment. In addition a post-procedural questionnaire could include a scale of 1-10 on how likely they are to use the stress ball at follow-up appointments.

## Conclusions

The results of this study found that the use of a stress ball as a distraction technique did not result in any significant reduction in stress levels in subjects undergoing scaling and root planing with local anesthetic.

## References

[1] Brahm CO, Lundgren J, Carlsson SG, Nilsson P, Corbeil J, Hagglin C (2012). Dentists' views on fearful patients. Problems and promises. Swed Dent J.

[2] Humphris G, King K (2011). The prevalence of dental anxiety across previous distressing experiences. J Anxiety Disord.

[3] Armfield JM, Heaton LJ (2013). Management of fear and anxiety in the dental clinic: a review. Aust Dent J.

[4] Oosterink FM, de Jongh A, Aartman IH (2008). What are people afraid of during dental treatment? Anxiety-provoking capacity of 67 stimuli characteristic of the dental setting. Eur J Oral Sci.

[5] Kain ZN, Mayes LC, Caldwell-Andrews AA, Karas DE, McClain BC (2006). Preoperative anxiety, postoperative pain, and behavioral recovery in young children undergoing surgery. Pediatrics.

[6] Rhudy JL, Meagher MW (2000). Fear and anxiety: divergent effects on human pain thresholds. Pain.

[7] Coriat IH (1946). Dental anxiety; fear of going to the dentist. Psychoanal Rev.

[8] Ruscheweyh R, Kreusch A, Albers C, Sommer J, Marziniak M (2011). The effect of distraction strategies on pain perception and the nociceptive flexor reflex (RIII reflex). Pain.

[9] Shenefelt PD (2013). Anxiety reduction using hypnotic induction and self-guided imagery for relaxation during dermatologic procedures. Int J Clin Exp Hypn.

[10] Appukuttan DP (2016). Strategies to manage patients with dental anxiety and dental phobia: literature review. Clin Cosmet Investig Dent.

[11] Ram D, Shapira J, Holan G, Magora F, Cohen S, Davidovich E (2010). Audiovisual video eyeglass distraction during dental treatment in children. Quintessence Int.

[12] Aydin D, Sahiner NC, Ciftci EK (2016). Comparison of the effectiveness of three different methods in decreasing pain during venipuncture in children: ball squeezing, balloon inflating and distraction cards. J Clin Nurs.

[13] Hudson BF, Ogden J, Whiteley MS (2015). Randomized controlled trial to compare the effect of simple distraction interventions on pain and anxiety experienced during conscious surgery. Eur J Pain.

[14] Tluczek A, Henriques JB, Brown RL (2009). Support for the reliability and validity of a six-item state anxiety scale derived from the State-Trait Anxiety Inventory. J Nurs Meas.

[15] Humphris GM, Morrison T, Lindsay SJE (1995). The Modified Dental Anxiety Scale: Validation and United Kingdom norms. Community Dental Health.

[16] Najafpour E, Asl-Aminabadi N, Nuroloyuni S, Jamali Z, Shirazi S (2017). Can galvanic skin conductance be used as an objective indicator of children's anxiety in the dental setting?. J Clin Exp Dent.

[17] Bekker HL, Legare F, Stacey D, O'Connor A, Lemyre L (2003). Is anxiety a suitable measure of decision aid effectiveness: a systematic review?. Patient Educ Couns.

[18] Melzack R (1999). From the gate to the neuromatrix. Pain.

[19] Tracey I, Mantyh PW (2007). The cerebral signature for pain perception and its modulation. Neuron.

[20] Legrain V, Crombez G, Mouraux A (2011). Controlling attention to nociceptive stimuli with working memory. PLoS One.

[21] Caprara HJ, Eleazer PD, Barfield RD, Chavers S (2003). Objective measurement of patient's dental anxiety by galvanic skin reaction. J Endod.

[22] Humphris GM, Dyer TA, Robinson PG (2009). The modified dental anxiety scale: UK general public population norms in 2008 with further psychometrics and effects of age. BMC Oral Health.

[23] Humphris GM, Freeman R, Campbell J, Tuutti H, D'Souza V (2000). Further evidence for the reliability and validity of the Modified Dental Anxiety Scale. Int Dent J.

[24] Julian LJ (2011). Measures of anxiety: State-Trait Anxiety Inventory (STAI), Beck Anxiety Inventory (BAI), and Hospital Anxiety and Depression Scale-Anxiety (HADS-A). Arthritis Care Res. (Hoboken).

[25] Marteau TM, Bekker H (1992). The development of a six-item short-form of the state scale of the Spielberger State-Trait Anxiety Inventory (STAI). Br J Clin Psychol.

[26] Tluczek A, Henriques JB, Brown RL (2009). Support for the reliability and validity of a six-item state anxiety scale derived from the State-Trait Anxiety Inventory. J Nurs Meas.

[27] Humphris GM, Hull P (2007). Do dental anxiety questionnaires raise anxiety in dentally anxious adult patients? A two-wave panel study. Prim Dent Care.

